# Stover Composition in Maize and Sorghum Reveals Remarkable Genetic Variation and Plasticity for Carbohydrate Accumulation

**DOI:** 10.3389/fpls.2016.00822

**Published:** 2016-06-08

**Authors:** Rajandeep S. Sekhon, Matthew W. Breitzman, Renato R. Silva, Nicholas Santoro, William L. Rooney, Natalia de Leon, Shawn M. Kaeppler

**Affiliations:** ^1^Department of Genetics and Biochemistry, Clemson UniversityClemson, SC, USA; ^2^Department of Agronomy, University of WisconsinMadison, WI, USA; ^3^DOE Great Lakes Bioenergy Research Center, University of WisconsinMadison, WI, USA; ^4^Institute of Mathematics and Statistics, Federal University of GoiásGoiânia, Brazil; ^5^Center for Chemical Genomics, University of MichiganAnn Arbor, MI, USA; ^6^Department of Soil and Crop Sciences, Texas A&M UniversityCollege Station, TX, USA

**Keywords:** sorghum, maize, biofuels, biofeedstocks, non-structural carbohydrates, structural carbohydrates

## Abstract

Carbohydrates stored in vegetative organs, particularly stems, of grasses are a very important source of energy. We examined carbohydrate accumulation in adult sorghum and maize hybrids with distinct phenology and different end uses (grain, silage, sucrose or sweetness in stalk juice, and biomass). Remarkable variation was observed for non-structural carbohydrates and structural polysaccharides during three key developmental stages both between and within hybrids developed for distinct end use in both species. At the onset of the reproductive phase (average 65 days after planting, DAP), a wide range for accumulation of non-structural carbohydrates (free glucose and sucrose combined), was observed in internodes of maize (11–24%) and sorghum (7–36%) indicating substantial variation for transient storage of excess photosynthate during periods of low grain or vegetative sink strength. Remobilization of these reserves for supporting grain fill or vegetative growth was evident from lower amounts in maize (8–19%) and sorghum (9–27%) near the end of the reproductive period (average 95 DAP). At physiological maturity of grain hybrids (average 120 DAP), amounts of these carbohydrates were generally unchanged in maize (9–21%) and sorghum (16–27%) suggesting a loss of photosynthetic assimilation due to weakening sink demand. Nonetheless, high amounts of non-structural carbohydrates at maturity even in grain maize and sorghum (15–18%) highlight the potential for developing dual-purpose (grain/stover) crops. For both species, the amounts of structural polysaccharides in the cell wall, measured as monomeric components (glucose and pentose), decreased during grain fill but remained unchanged thereafter with maize biomass possessing slightly higher amounts than sorghum. Availability of carbohydrates in maize and sorghum highlights the potential for developing energy-rich dedicated biofuel or dual-purpose (grain/stover) crops.

## Introduction

Grasses have played a key role in supporting life across the globe by providing calories derived primarily from cereal grains for humans and forages for animal feed. With increasing world population, decreasing arable land and fresh water, and emerging demand for plant-based fuels, grasses are poised for yet another major role in sustaining human life on earth. A unique property that makes grasses ideal to meet this challenge is their potential to accumulate large amount of carbohydrates stored in the vegetative plant parts or stover (Widstrom et al., 1984; Hoffmann-Thoma et al., 1996; Ruuska et al., 2006; White et al., 2011). The carbohydrates in stover exist both as structural components, composed of insoluble polysaccharides—cellulose and hemicellulose—incorporated into the cell wall, and as non-structural storage molecules comprised of soluble sugars or sugar polymers primarily stored in the parenchyma cells. Grasses contribute ~74% (~2.4 billon metric tons/year) of stover, plant biomass leftover after harvesting grain, worldwide (Lal, 2005) which has traditionally served as a key animal feed resource and widely used as roughage especially when supply of fodder or other forms of feed is low (Owen and Jayasuriya, 1989; Parthasarathy Rao and Hall, 2003; Hellin et al., 2013). Grass stems are considered to be transient sinks of non-structural carbohydrates that act as a “buffering system” between the primary source (leaves) and primary (terminal) sink during various developmental and environmental transitions (Schnyder, 1993; Blum, 1998; Ruuska et al., 2006; Mir et al., 2012; Slewinski, 2012). Given that cereal crops are typically bred for grain that acts as the primary sink, stover—the secondary sink—is considered to have low nutrient and energy content. However, with increased demand for stover as a source of renewable energy, development of dual-purpose crops which, in addition to grain, provide high quality stover need to be developed.

Maize (*Zea mays* L.) and sorghum (*Sorghum bicolor* L. Moench), two key grasses that employ C_4_ photosynthesis and thus exhibit higher carbon fixation and water use efficiency, have excellent potential to supply stover, especially in hotter and drier climates. Maize is one of the most important cereals in the world accounting for an average production of 840 million metric tons/year in the last decade (FAOSTAT, 2014). Considering an average harvest index of 50%, a similar proportion of stover productivity can potentially be achieved thus making maize the most abundant source of cereal stover. Maize was domesticated in the subtropics primarily for use as a grain. Current maize hybrids grown in temperate U.S. environments have been primarily selected for either grain or a combination of grain and biomass (silage). Day-length sensitive tropical maize hybrids however, flower late when grown in a temperate long-day regime, producing little or no grain, and greater amounts of vegetative biomass than commercial temperate hybrids (White et al., 2011). Breeding efforts focusing on maize as a source of grain have a substantially longer history than efforts focused on using its biomass. Furthermore, research related to the concentration and genetic variability of polysaccharides in maize biomass has focused almost entirely on development of genotypes dedicated to production of silage, which consists of a mixture of grain and stover. Genetic variation for accumulation of polysaccharides in stover (secondary sink) of maize germplasm developed specifically for grain, however, has not been systematically examined and compared to germplasm developed for stover use. Sorghum accounts for ~3% of total grain production worldwide (Erenstein et al., 2011). With its impressive genetic variability, sorghum can be tailored to different environments and biomass processing systems including sweet sorghum for high amounts of extractable sucrose from the stem, and biomass or energy sorghum for high total lignocellulosic biomass yield (Rooney, 2004). Due to its potential for high total biomass production (particularly for energy sorghum), high accumulation of fermentable sugars in the culm (for sweet sorghum), resistance to biotic and abiotic stress, and ability of the crop to thrive in low fertility environments (Shoemaker and Bransby, 2010), sorghum is perceived as an excellent biofuel crop. Like maize, however, availability of structural and non-structural polysaccharides have not been compared among sorghum genotypes developed for distinct end uses in a single study.

To fully realize the advantage of C_4_ photosynthesis in maize and sorghum, focus should be shifted to allocation of high amounts of photosynthate to multiple harvestable organs thus developing multipurpose crops. To learn about the extent and developmental dynamics of carbohydrate accumulation in vegetative tissues, we examined the accumulation of various polysaccharides in different maize and sorghum plant types (grain, forage, biomass etc.) during the last ~8 weeks of the life cycle. The goal of this study was to generate baseline information for supporting the energy densification efforts in sorghum and maize in secondary sinks by converting excess carbohydrates into novel molecules like vegetative oils. Specifically, we set out to understand the extent and nature of carbohydrates partitioned to stover during the period leading up to physiological maturity as those are of great consequence for development and utilization of secondary sinks. Given the increased demand for carbohydrates for food and energy needs, this information will be useful for devising strategies to develop energy-dense dual-purpose (grain/biomass) or dedicated biomass crops for biofuels.

## Materials and methods

### Plant materials, growing conditions, and sampling details

Maize and sorghum hybrids listed in Table 1 were grown at the West Madison Agricultural Research Station, Verona, WI during summer of 2012 in a randomized complete block design with two replications. Each plot consisted of one row of 2.9 m in length with row spacing of 76 cm and plants spaced 24 cm apart. The experimental field has Plano silt loam soil with pH 6.8, 3% organic matter, 93 ppm phosphorus, and 240 ppm potassium. During field preparation, 200 kg/acre of Urea (46-0-0) was applied. The herbicides Callisto (Syngenta, Greensboro, NC; 142 g per acre), Dual II (Syngenta, Greensboro, NC; 710 ml per acre), and Simazine (Agrisolutions, Brighton, IL; 227 g per acre) were applied 1 day after planting.

**Table 1:** **List and description of genotypes included in the study**.

	**Genotype**	**Abbreviation**	**Crop**	**Type/end-use**
1	F2F626	Zm_1	Maize	Brown mid-rib3 (BMR) forage
2	F2F665	Zm_2	Maize	Brown mid-rib3 (BMR) forage
3	5200	Zm_3	Maize	Biomass
4	A6323GT3	Zm_4	Maize	Biomass
5	LG2642VT3	Zm_5	Maize	Long-season silage (115 d) ^‡^
6	MC590	Zm_6	Maize	Long-season silage (116 d) ^‡^
7	(CL-RCW104/CML494)//CML498	Zm_7	Maize	Tropical
8	(CL-RCW109/CML494)//CML498	Zm_8	Maize	Tropical
9	(CL-RCW110/CML494)//CML498	Zm_9	Maize	Tropical
10	(CL-RCW43/CL-RCW42)//CML269	Zm_10	Maize	Tropical
11	(CL-RCW83/CML494)//CML269	Zm_11	Maize	Tropical
12	(CL-RCW85/CML494)//CML269	Zm_12	Maize	Tropical
13	(CL-RCW86/CML494)//CML269	Zm_13	Maize	Tropical
14	(CL-RCW94/CML494)//CML269	Zm_14	Maize	Tropical
15	(CL-RCW98/CML494)//CML269	Zm_15	Maize	Tropical
16	(CL-RCW99/CML494)//CML269	Zm_16	Maize	Tropical
17	34A89	Zm_17	Maize	Grain
18	P0448R	Zm_18	Maize	Grain
19	ATx645/BTx2752//RTx2785	Sb_1	Sorghum	Photoperiod insensitive forage
20	ATx631/RTx2910	Sb_2	Sorghum	Photoperiod sensitive forage (12.25 h) ^¥^
21	ATx631/RTx2909	Sb_3	Sorghum	Photoperiod sensitive forage(12.25 h) ^¥^
22	ATx645/BTx2752//R10764	Sb_4	Sorghum	Very photoperiod sensitive biomass (< 12 h) ^¥^
23	ATx645/BTx2752//R10788	Sb_5	Sorghum	Very photoperiod sensitive biomass (< 12 h) ^¥^
24	TAMX08001	Sb_6	Sorghum	Very photoperiod sensitive biomass (< 12 h) ^¥^
25	TX09055	Sb_7	Sorghum	Mildly photoperiod sensitive sweet (< 13 h) ^¥^
26	TX09052	Sb_8	Sorghum	Photoperiod insensitive sweet
27	TX09062	Sb_9	Sorghum	Photoperiod sensitive sweet (12.25 h) ^¥^
28	ATx2928/RTx436	Sb_10	Sorghum	Photoperiod insensitive mid-season grain
29	ATx2752/RTx437	Sb_11	Sorghum	Photoperiod insensitive mid-season grain
30	ATx645/RTx2783	Sb_12	Sorghum	Photoperiod insensitive mid to full-season grain

‡ , days (d) to maturity;

¥ *, Threshold (hours, h) of daylight for triggering photoperiod sensitivity*.

Plants were allowed to open pollinate. The leaf and internode tissue samples were collected at 65, 95, and 120 days after planting (DAP). At each stage, sample collection was done in a single session between 8:30 a.m. and 12:00 p.m. For each replication, the samples were pooled from two competitive (surrounded by optimally spaced neighboring plants on both sides), randomly chosen plants. Leaf samples were composed of a 30 cm section of leaf blade and mid-rib, excluding ligule, and sheath, from the leaf below the sixth elongated internode of the main stem if plants had tillers. The internode samples comprised the entire sixth elongated internode excluding the nodes. All samples were chopped into small pieces, flash frozen in liquid nitrogen, and stored at −80°C. All the biochemical analyses were performed on pooled tissue from two competitive plants per plot.

### Metabolic analysis

Accumulation of major carbohydrates including cell wall-bound glucose and pentose, free glucose, sucrose, and starch were measured in the internode and leaf tissue from frozen tissues. Most of these assays have been previously described (Santoro et al., 2010; Sekhon et al., 2012). The frozen samples were lyophilized and finely ground on a custom-designed robot (Santoro et al., 2010). For each sample, 1.4 mL tubes containing 1.3–1.7 mg of the ground tissue were prepared for each assay. Each sample was measured twice and the average of the two technical replicates was used for statistical analyses.

For measuring glucose and pentose monomers in the cell wall, samples were pre-treated (Santoro et al., 2010) and digested with a 50 μL solution containing 0.5 μL Accellerase 1000 (Genencor, Rochester, NY), 1 M citrate buffer (pH 4.5), and 0.01% sodium azide to give a final volume of 0.8 ml and a final enzyme concentration of 50 mg protein/g glucan. After incubation at 50°C for 20 h with rotation, supernatant was collected by centrifugation at 1500 × g for 3 min. Glucose content was assayed using the glucose oxidase/peroxidase (GOPOD) method (K-GLUC, Megazyme, Ireland) as described (Santoro et al., 2010). Each digestion assay was quantitated for glucose and pentose in quadruplicate. Glucose and pentose yield refers to the amount of each sugar released relative to the total dry mass dispensed into each tube.

For assaying free glucose, 750 μL of distilled water was pipetted to the pre-prepared tubes followed by addition of 50 μL of a solution containing 1 M citrate buffer (pH 4.5) and 0.01% sodium azide resulting in a final volume of 0.8 mL. Subsequent sample incubation and processing was identical to that used for the digestibility assay. Glucose was assayed using the GOPOD method described above.

For determination of sucrose, samples were pre-treated with 6.25 mM NaOH, heated to 90°C for 3 h, and cooled as described earlier (Cass et al., 2015). The combination of alkaline environment and high heat during this pre-treatment step are sufficient to degrade all pre-existing free glucose (Moulik et al., 1978; Yang and Montgomery, 2007). The pre-treatment was followed by addition of 47.3 μL of a solution containing 4 μL Invertase (1 U/μL; Sigma-Aldrich, St. Louis, MO) in 30 mM citrate buffer (pH 4.5) and 0.01% sodium azide to each tube. The rest of the processing of these samples was identical to that used for the glucose and pentose assay described above. Thus, all the glucose present after invertase treatment is derived from hydrolysis of sucrose. The amount of sucrose was obtained by doubling the amount of glucose determined.

For determination of starch, 150 μL of 2 M KOH was added to the pre-prepared sample tubes followed by incubation at 90°C for 1 h and cooling on ice and then the solution was neutralized by adding 600 μL 1.2 M NaOAc buffer (pH 3.8). For starch hydrolysis, 60 μL of a solution containing 5 μL amyloglucosidase (K-TSTA, Megazyme, Ireland), 5 μL α-amylase (K-TSTA, Megazyme), distilled water, and 0.01% sodium azide was added. The rest of the processing of these samples was identical to that used for the glucose and pentose assay described above. The heating before starch hydrolysis is sufficient to degrade all pre-existing free glucose in the samples, thus, glucose detected after the hydrolysis step is derived from starch. Glucose was assayed using the GOPOD method described above.

### Statistical analysis

To verify the hypothesis that the effect of interaction between genotype and sampling stages is significant, analyses of variance (ANOVA) were done for each form of carbohydrates (sucrose, free glucose, cell wall-bound glucose, cell wall-bound pentose, and starch) measured on each tissue (internode and leaf). The statistical model used was:

$$\begin{array}{l} y_{ijk}=\mu +g_{i}+s_{j}+{\left(gs\right)}_{ij}\;+\;b_{k}\;+\;\varepsilon_{ijk} \end{array}$$

where *y*_*ijk*_ is the observed response variable of *i-th* genotype evaluated at the *j-th* sampling stages and *k-th* replication; μ is the intercept; *g*_*i*_ is the effect of the *i-th* genotype; *b*_*k*_ is the effect of the *k-th* replication; *s*_*j*_ is the effect of the *j-th* sampling stages; (*gs*)_*ij*_ is the interaction between *i-th* genotype and *j-th* sampling stages and ε_*ijk*_ is a random error of model. The sampling stages were (65, 95, and 120 DAP) and all effects were considered fixed. To test the hypothesis of differences among genotypes within each of the sampling stages, ANOVA were performed considering the effect of genotype nested within sampling stages. ANOVA, rank correlation and all other computations were performed using the statistical software R (R Core Team, 2014). Tukey test in R package agricolae was used for multiple comparison of hybrids for different sugars.

## Results

### Range of phenology in sorghum and maize hybrids is reflected in patterns of carbohydrate accumulation and partitioning

**Figure 1:**
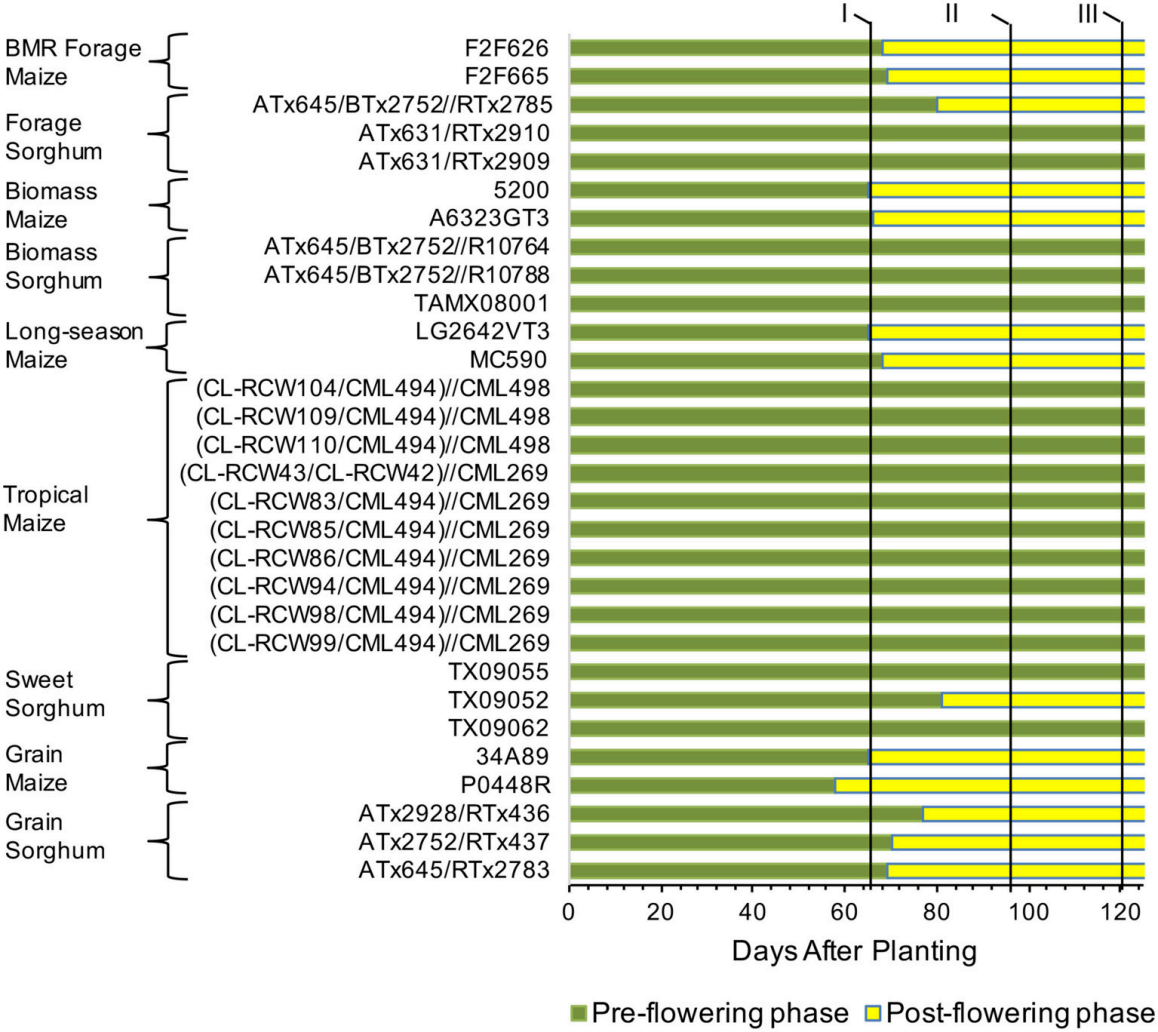
**Transition from vegetative to reproductive stages and sampling timing for maize and sorghum hybrids included in the study**. Flowering data represents average of both field replications. The pre- and post-flowering stages were determined based on growth pattern of a prototypical maize grain hybrid (34A89). The flowering data was not recorded after 95 DAP stage and those hybrids not flowering at that stage were considered as non-flowered. Such hybrids did not produce any seed. Roman numerals at the top and the vertical lines next to the numerals indicate three sampling stages, I, 65 DAP; II, 95 DAP; III, 120 DAP.

To get a detailed picture of carbohydrate accumulation and partitioning in different plant types of sorghum and maize, we examined hybrids suited for distinct end uses (Table 1). Maize hybrids included those developed for grain yield, forage digestibility achieved in part by introgression of the *brown midrib 3* (BMR) mutation, biomass yield, tropical (photoperiod sensitive), and long-season hybrids expected to provide high biomass yield due to prolonged vegetative growth. Sorghum hybrids included those developed for grain, forage, biomass yield, and extractable stem sucrose (sweet sorghum). Differences in the phenology of these hybrids were evident from variation in flowering and, while all the temperate maize and the grain sorghum hybrids flowered in the test environment, the tropical maize and most of the forage, biomass, and sweet sorghum hybrids either did not flower or flowered too late to produce any seed (Figure 1).

Given differences in phenology and inherent differences in growth patterns of sorghum and maize, sampling stages were determined following a prototypical maize growth pattern represented by maize commercial hybrid 34A89. The three sampling stages included: (1) 65 DAP denoting near-completion of vegetative growth of the adapted maize hybrids and of the primary tiller of grain sorghum, and initiation of flowering in the majority of the hybrids; (2) 95 DAP representing completion of vegetative growth of maize and that of most sorghum tillers, and near-completion of grain fill in the majority of hybrids that flowered in the test environment; and (3) 120 DAP signifying physiological maturity of the feedstocks (Figure 1). Furthermore, due to the differences among hybrids noted above, dissimilarities in levels of various carbohydrates may exist among different internodes and leaves within a plant. We previously reported that, at the 95 DAP stage of the inbred line B73, the second through sixth elongated internodes had comparable levels of glucose and pentose (Muttoni et al., 2012). Therefore, while it may not be possible to sample identical tissues for all the hybrids, a specific internode (sixth internode above ground) and a specific leaf (below the sixth internode) were selected for sake of consistency in sampling. Furthermore, sampling sixth internode, which was consistently below the ear node in all maize genotypes examined, circumvented the possible confounding effect of the ear position on the composition of the sampled internode. For all metabolites in each tissue, significantly high rank correlations were observed between the two biological replicates (Table 2). For most forms of the sampled carbohydrates, significant genotypic differences were observed within each of the sampling stages for both tissues, with the exception of starch accumulation in leaves (Table 2).

**Table 2:** **Significance of variance components in analysis of variance for accumulation of various carbohydrates for internodes and leaves for all maize and sorghum hybrids evaluated in this study. Also shown, in parenthesis, are rank correlations between two biological replicates for each metabolite in each tissue**.

	**P(>F)**				
**Source**	**Free glucose**	**Sucrose**	**Glucose**	**Pentose**	**Starch**
**INTERNODES**					
Rep	NS (0.54^***^)	NS (0.58^***^)	^***^ (0.76^***^)	^**^ (0.76^***^)	NS (0.69^***^)
Stage	^***^	^***^	^***^	^***^	^***^
Stage:hybrid	^***^	^***^	^***^	^***^	^***^
Stage:hybrid: 65 DAP	^***^	^**^	^***^	^***^	NS
Stage:hybrid: 95 DAP	^*^	^***^	^***^	^***^	NS
Stage:hybrid: 120 DAP	NS	^***^	^***^	^***^	^***^
**LEAVES**					
Rep	NS (0.57^***^)	^**^ (0.51^***^)	^*^ (0.58^***^)	^*^ (0.44^***^)	^***^ (0.34^**^)
Stage	^***^	^***^	^***^	^***^	^***^
Stage:hybrid	^***^	^***^	^***^	^***^	NS
Stage:hybrid: 65 DAP	NS	NS	^***^	^***^	NS
Stage:hybrid: 95 DAP	^**^	^***^	^***^	^***^	NS
Stage:hybrid: 120 DAP	^***^	^***^	^***^	^***^	^***^

* p = 0.05;

** p = 0.01;

*** *p = 0.001; NS, non-significant; rep, replication; stage, growth stage; DAP, days after planting*.

### Accumulation of non-structural carbohydrates across developmental gradients corresponds with end use of the hybrids

**Figure 2:**
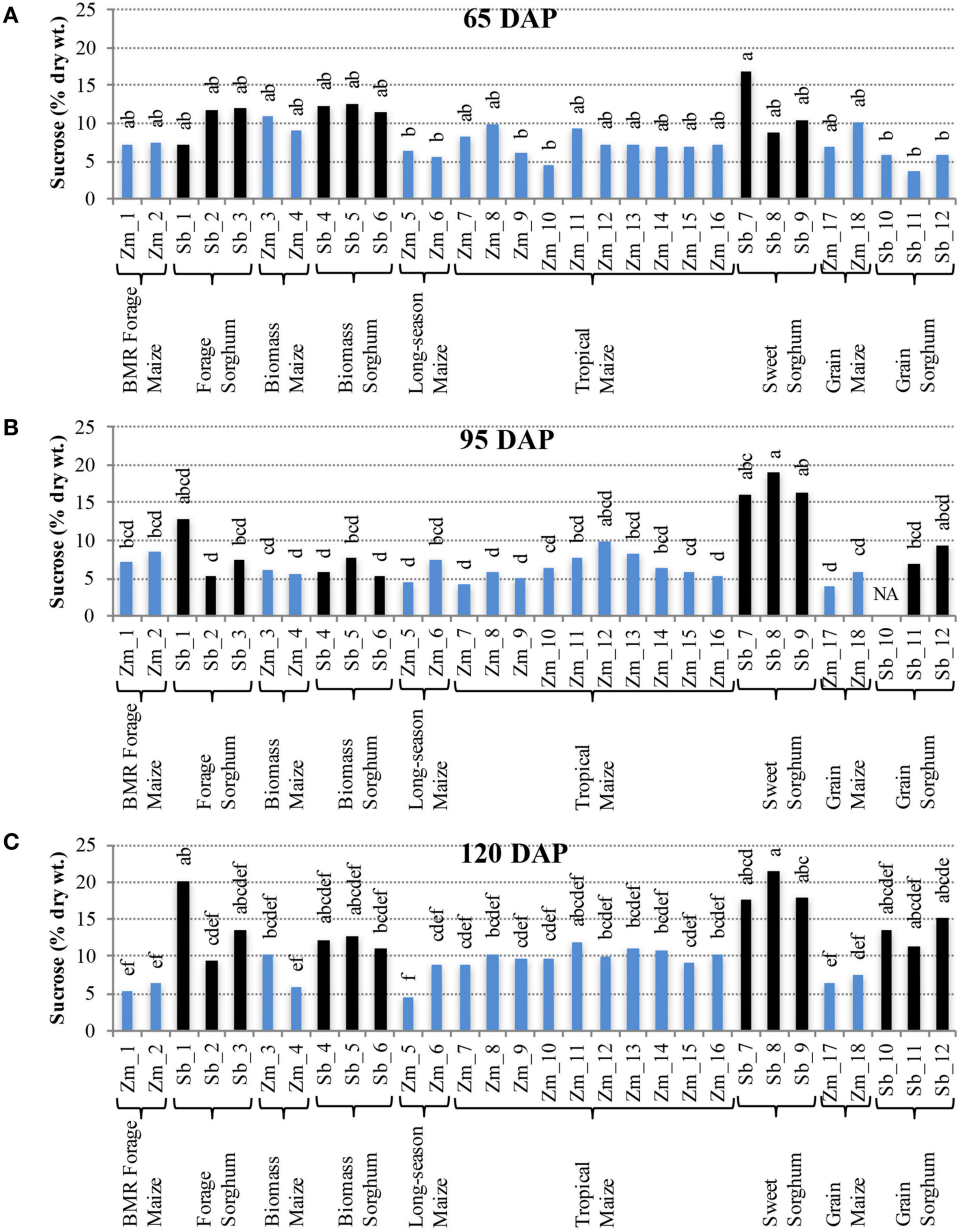
**Sucrose accumulation in internodes of diverse maize and sorghum hybrids at the 65 DAP (A), 95 DAP (B), and 120 DAP (C) stages**. NA, no data available. Different letters on bars represent statistically significant differences (*P* < 0.05) for a given stage.

Distinct levels of two of the non-structural carbohydrates, sucrose, and free glucose, were observed in hybrids developed for different end uses. For accumulation of sucrose at 65 DAP, most of the maize and sorghum hybrids had similar levels with the internode sucrose levels varying between 4–11% for maize and 3–17% for sorghum (Figure 2A). However, at 95 DAP, higher genotypic variability for internode sucrose was observed in both maize and sorghum hybrids (Figure 2B). Sorghum had greater variability (5–19%) with sweet sorghum possessing higher sucrose, while maize had relatively less variability (4–10%) with tropical and non-flowering hybrids accumulating higher amounts. These trends were preserved at physiological maturity (120 DAP) and, in general, a modest increase in sucrose content was observed for sorghum (9–21%) and maize (5–12%; Figure 2C). In leaves, sucrose levels were relatively low compared to internodes throughout the stages examined and ranged between 1–6% in maize and 4–9% in sorghum hybrids at 120 DAP (Figure S1).

**Figure 3:**
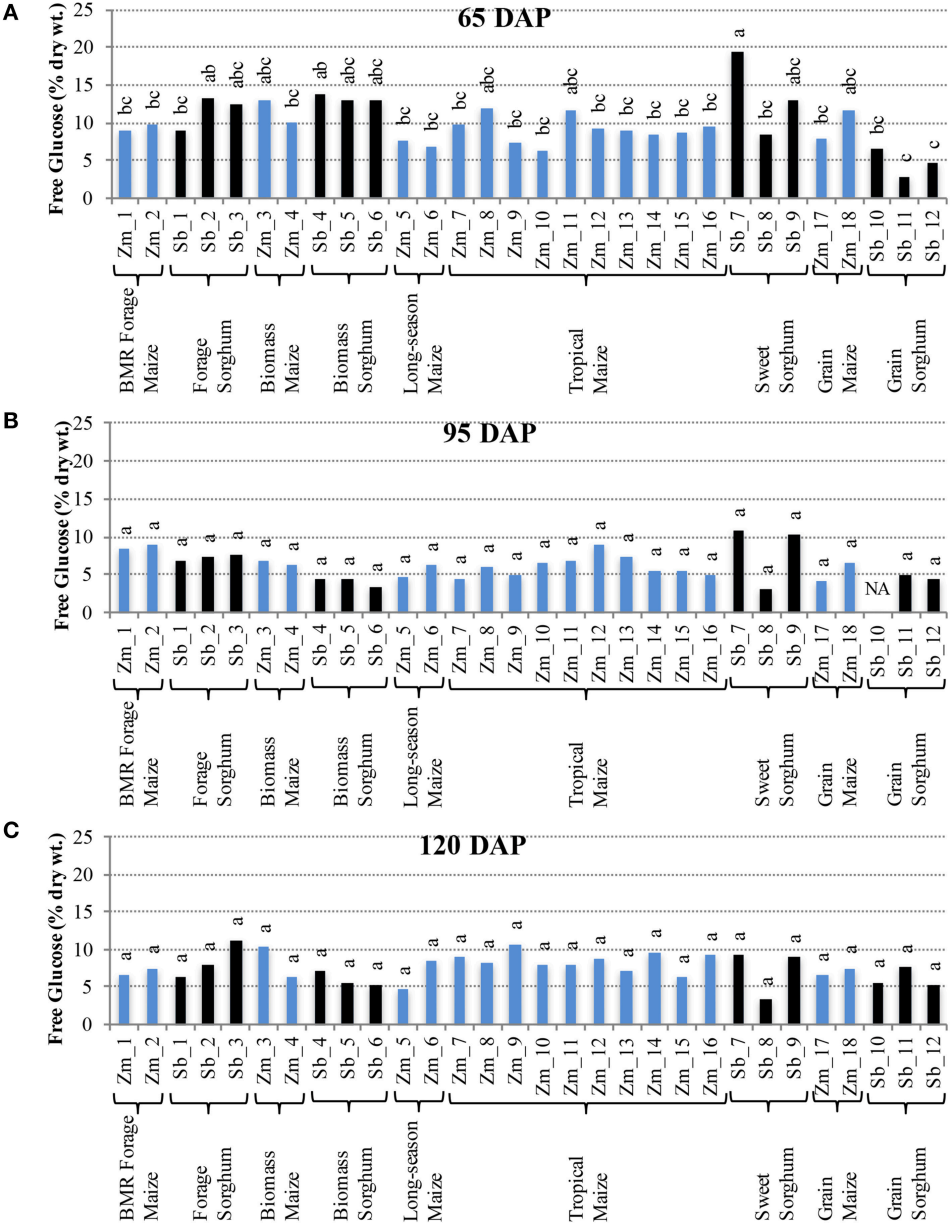
**Free glucose accumulation in internodes of diverse maize and sorghum hybrids at the 65 DAP (A), 95 DAP (B), and 120 DAP (C) stages**. NA, no data available. Different letters on bars represent statistically significant differences (*P* < 0.05) for a given stage.

Interestingly, the trends for accumulation of free glucose in internodes were in contrast with those of sucrose. More variation among hybrids was observed for free glucose at 65 DAP (Figure 3A), but these differences were not observed at 95 and 120 DAP (Figures 3B,C). At 65 DAP, the amounts of free glucose ranged between 6–13% for maize and 3–19% for sorghum. At 95 DAP, the range narrowed to 4–9% in maize and 3–11% for sorghum (Figure 3B), and the range remained similar at 120 DAP except a slightly higher upper limit (5–11%) was observed for maize (Figure 3C). In leaves, however, higher genotypic differences were observed for free glucose levels at 120 DAP which varied between 1–7% for maize and 4–11% for sorghum (Figure S2).

Levels of starch, another non-structural carbohydrate, in internodes were very low at 65 DAP (0–1%) and 95 DAP (0–3%) for both sorghum and maize (Figures S3A,B). However, higher variation was observed at 120 DAP (0–9%) with a substantially higher accumulation observed in sorghum (2–9%) compared to maize (0–2%; Figure S3C). Starch accumulation in leaves was generally quite low (< 2%) across all hybrids and developmental stages and did not show major fluctuations (data not shown).

It is important to note that sizeable, and often significant, differences for accumulation of non-structural carbohydrates were observed among hybrids developed for a particular purpose (e.g., forage sorghum, tropical maize etc.; Figures 2, 3). Additionally, when examining the changes in accumulation of two major non-structural carbohydrates, sucrose and free glucose, in internodes across the post-flowering developmental period, a general trend of hyperaccumulation around flowering, depletion during the period coinciding with rapid grain fill, and re-accumulation during physiological maturation was observed (Figures 2, 3). However, as noted above, these trends were not universal and outliers were observed even within each species and within each group of hybrids developed for a particular purpose.

### Structural polysaccharide content is determined early during development and remains stable during physiological maturation

Grass cell wall structural polysaccharides, cellulose (consisting entirely of glucose monomers), and hemicellulose (composed of pentose and glucose) (Vogel, 2008), are important sinks for photosynthate. To examine the dynamics of photosynthate partitioning to these polysaccharides, we measured changes in glucose and pentose content in cell walls during the three key developmental stages.

**Figure 4:**
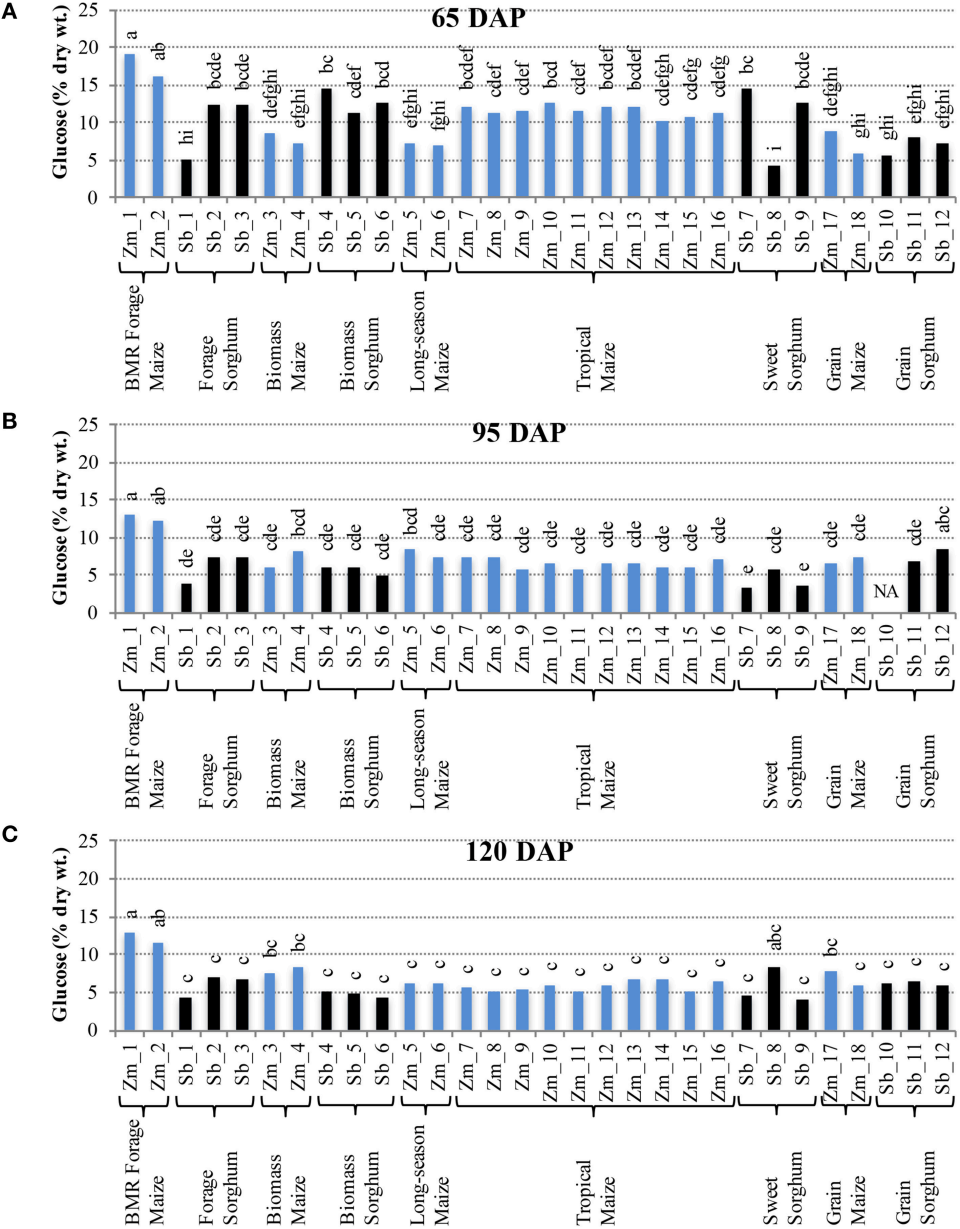
**Glucose accumulation in internodes of diverse maize and sorghum hybrids at the 65 DAP (A), 95 DAP (B), and 120 DAP (C) stages**. NA, no data available. Different letters on bars represent statistically significant differences (*P* < 0.05) for a given stage.

**Figure 5:**
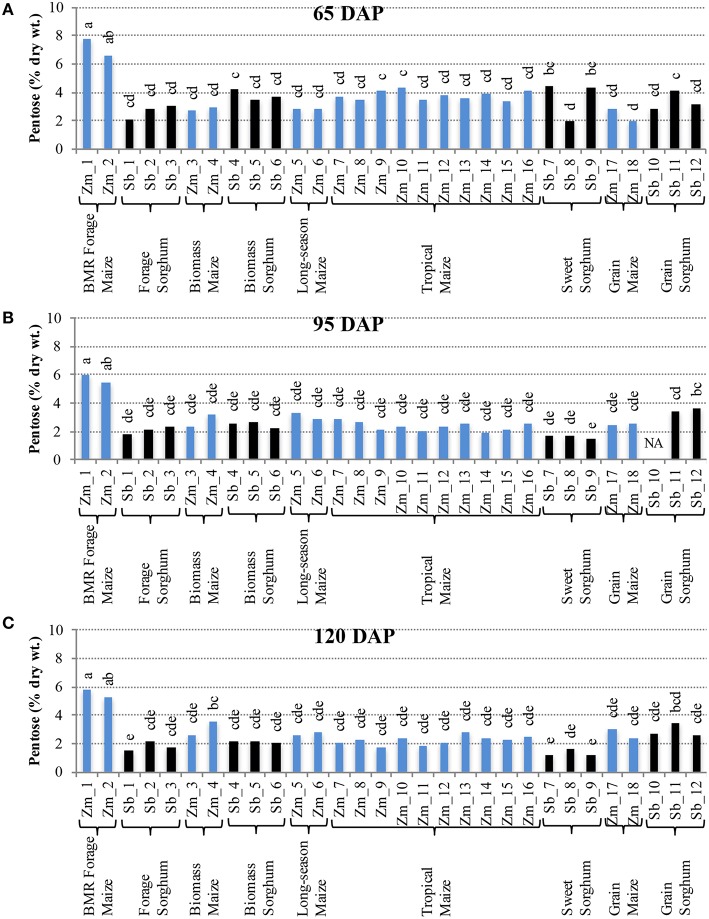
**Pentose accumulation in internodes of diverse maize and sorghum hybrids at the 65 DAP (A), 95 DAP (B), and 120 DAP (C) stages**. NA, no data available. Different letters on bars represent statistically significant differences (*P* < 0.05) for a given stage.

Comparison of individual hybrids for internode glucose content showed significant differences among hybrids during the early stage (65 DAP) (Figure 4A), but relatively lower variation at later stages (95 and 120 DAP; Figures 4B,C). At 65 DAP, glucose levels in internodes ranged between 6–19% for maize and 4–14% for sorghum. At 95 DAP, these levels generally decreased and ranged between 6–13% for maize and 3–8% for sorghum hybrids and remained in a similar range at 120 DAP. Glucose content in leaves was generally higher than in the internodes at each stage and ranged between 9–17% for maize and 9–12% for sorghum at the 120 DAP stage (Figure S4). The amount of pentose in internodes ranged between 2–8% for maize and 2–4% for sorghum at 65 DAP and this range was mostly unchanged at 95 and 120 DAP (Figure 5). Leaves had slightly higher amounts of pentose when compared to internodes in maize (3–8%) and sorghum (2–4%; Figure S5). Consistent with lower lignification of its cell walls (Barrière et al., 1994), BMR maize hybrids had significantly higher amounts of extractable glucose and pentose in the internodes while all other maize and sorghum hybrids were statistically indistinguishable for these cell wall-bound sugars.

## Discussion

Understanding the regulation and partitioning of carbohydrates is key to enhance productivity in major cereal crops including wheat (*Triticum aestivum* L.; Blum, 1998; Ruuska et al., 2006), maize (Braun et al., 2006, 2014; Bihmidine et al., 2013), barley (*Hordeum vulgare* L.; Schnyder, 1993), and sorghum (Slewinski, 2012; Bihmidine et al., 2015). While substantial information has been generated for carbohydrate content in the primary sink for maize and sorghum genotypes developed for a specific end use, availability and genetic variation of carbohydrates in the secondary sinks of those genotypes has not been explored. Herein, we examined the accumulation of selected non-structural (free glucose, sucrose, starch) and structural (glucose, pentose) carbohydrates in stover, both as a primary and a secondary sink, in maize and sorghum hybrids developed for distinct end uses. While other carbohydrate and non-carbohydrate metabolites are also of potential interest, those were not examined in the current study, as the focus was on key metabolites of relevance for biofuel and genetic engineering strategies.

Non-structural carbohydrates are attractive targets for the biofuel industry due to easy and energy efficient extraction, and conversion into energy molecules as demonstrated by successful utilization of sugarcane stem sucrose for ethanol production. Therefore, the presence of large amounts of non-structural carbohydrates, specifically sucrose and free glucose, in internodes of maize (9–21%) and sorghum (16–27%) at physiological maturity is of great significance for the biofuel industry. High genetic variation for carbohydrate accumulation in the maize internodes observed here and elsewhere (Van Reen and Singleton, 1952; Daynard et al., 1969; White et al., 2011), together with high heritability of this trait (Widstrom et al., 1984), highlights the feasibility of developing “sweet maize” similar to sweet sorghum. Indeed, such efforts in the past have resulted in development of maize hybrids with high sucrose (Singleton, 1948; Marten and Westerberg, 1972) and, in some cases, the stalk anatomy of such maize genotypes resembles that of sorghum and sugarcane rather than traditional maize genotypes (Singleton, 1948). Substantial amounts (15–18%) of these carbohydrates in stems of grain maize and grain sorghum hybrids highlight the potential for developing dual-purpose (grain/stover) crops that will support both grain fill and high stem sugar content. In such a system, the grain can be used for human/animal consumption while the energy dense stover can further be utilized as a biofuel or animal feedstock. Since breeding programs focusing on grain yield almost never directly focus on content of non-structural carbohydrates in the stover, mere inclusion of this trait as a selection criterion could further enhance the energy density of the stover. This approach has been implemented for the development of sorghum cultivars (Blümmel and Reddy, 2006; Ganesamurthy et al., 2012). The current study highlights the potential of this approach in grain maize, which has tremendous grain productivity but has not received much attention for stover improvement. It should be noted that fructose is also present in maize and sorghum stover (Chen et al., 2013; Han et al., 2013; Matsakas and Christakopoulos, 2013), however, this particular non-structural carbohydrate was not assayed in the current study.

In maize hybrids the non-structural carbohydrates, specifically sucrose, showed a modest accumulation during physiological maturation (120 DAP) compared to sorghum (Figures 2, 3). This is consistent with a progressively weakening sink demand after grain filling, especially in the genotypes developed for grain yield. In most sorghum hybrids, however, significantly higher levels of non-structural carbohydrates (particularly sucrose) accumulated at physiological maturation. This sustained assimilation of photosynthates in sorghum indicates the ability of this grass species to remobilize sugars to multiple sinks resulting in higher net carbon yield. Reduced sink demand and ensuing accumulation of non-structural carbohydrates in the leaf cells undermines photosynthesis via feedback inhibition thereby inducing premature senescence (Christensen et al., 1981; Crafts-Brandner et al., 1984; Ceppi et al., 1987; Wingler and Roitsch, 2008; Sekhon et al., 2012). Conversely, steady or enhanced remobilization of these excess carbohydrates from source to sink tissue in the stem will help in maintaining photosynthetic assimilation (Pollock et al., 2003). Furthermore, immobilization and densification of the non-structural carbohydrates into novel energy molecules can further increase sink potential. For instance, *de novo* synthesis of a non-degradable sucrose isomer (isomaltulose) in the vacuoles of sugarcane delayed senescence by 15–20 days, enhanced photosynthesis, elevated sucrose transport and sink strength, and doubled the total sugar content (Wu and Birch, 2007). Converting these carbohydrates to energy molecules has also been demonstrated in Arabidopsis (*Arabidopsis thaliana*), wherein, ectopic accumulation of triacylglycerols in vegetative plant parts was achieved by overexpression of transcription factor WRINKLED1 involved in seed oil biosynthesis (Sanjaya et al., 2011). There has also been considerable interest in enhancement of mixed-linkage glucans in grasses that serve as energy storage and are generally found in low amounts in grass cell walls (Buckeridge et al., 2004; Falter et al., 2015; Loqué et al., 2015). Variation for accumulation of non-structural carbohydrates in sorghum and maize stover supports biotechnological approaches for energy densification and development of dual-purpose (grain/stover) crops. Using a comparative genomics approach to understand the molecular basis of such variation in these two closely related C_4_ grasses will be a key to this endeavor.

Decrease in amounts of structural polysaccharides during the period between 65 and 95 DAP likely represents increased lignification and crosslinking mediated by ferulic and *p*-coumaric acid (Jung and Casler, 2006). Higher amounts of glucose and pentose in the maize BMR hybrids, for instance, are the result of mutations in the lignin biosynthetic pathway (*brown midrib 1* and *brown midrib 3*) that allow easy accessibility of these carbohydrates (Barrière et al., 1994). However, lack of major fluctuations in structural polysaccharides for each of the hybrids, particularly during and after grain fill (95 and 120 DAP), is in distinct contrast to the generally higher variability and higher levels of non-structural carbohydrates at those stages. Therefore, excess photosynthate produced during the period leading up to physiological maturation appears to be preferentially stored as non-structural carbohydrates. While structural polysaccharides, particularly cellulose, are difficult to extract due to recalcitrance of the lignocellulose biomass to hydrolysis (Himmel et al., 2007), preferential accumulation of non-structural carbohydrates can help in designing carbon neutral feedstocks. It should, however, be noted that trends of carbohydrate partitioning in photoperiod sensitive genotypes included in this study are valid only in temperate environments. These genotypes could substantially change in their native environment due to flowering and ensuing activity of the grain sink.

In summary, a survey of a small yet representative number of sorghum and maize genotypes demonstrates remarkable variation for carbohydrate content in stover existing as the secondary sink. Furthermore, non-structural carbohydrates appear to be the preferred kind of carbohydrates accumulating in stover near physiological maturity, and relatively easy extraction of these carbohydrates supports the feasibility of dual purpose crop plants. Maize is currently used as a dual-purpose grain/biofuel crop by harvesting the grain and then collecting stover by baling, or selectively collecting cobs during grain harvest at a small scale. Enhancing the non-structural carbohydrates and converting those into energy dense and easily extractable molecules can further enhance the scope of maize as a dual-purpose crop. Modulating the carbohydrate partitioning from primary sinks to secondary sinks later in development should result in an overall increase in carbohydrate yield in maize and sorghum.

## Author contributions

SK, ND, RSS designed the research; RSS, MB, NS performed the experiments, RSS and RRS did the data analysis; WR provided materials; RSS, WR, ND, SK wrote the manuscript.

## Funding

This work was supported by the U. S. Department of Energy Great Lakes Bioenergy Research Center (Department of Energy Office of Science BER DE-FC02-07ER64494). RSS is supported by Clemson University.

### Conflict of interest statement

The authors declare that the research was conducted in the absence of any commercial or financial relationships that could be construed as a potential conflict of interest.

## Supplementary material

Figure S1 **Sucrose accumulation in leaves of diverse maize and sorghum hybrids at the 65 DAP (A), 95 DAP (B), and 120 DAP (C) stages**. Different letters on bars represent statistically significant differences (*P* < 0.05) for a given stage.

Figure S2 **Free glucose accumulation in leaves of diverse maize and sorghum hybrids at the 65 DAP (A), 95 DAP (B), and 120 DAP (C) stages**. Different letters on bars represent statistically significant differences (*P* < 0.05) for a given stage.

Figure S3 **Starch accumulation in internodes of diverse maize and sorghum hybrids at the 65 DAP (A), 95 DAP (B), and 120 DAP (C) stages**. NA, no data available. Different letters on bars represent statistically significant differences (*P* < 0.05) for a given stage.

Figure S4 **Glucose accumulation in leaves of diverse maize and sorghum hybrids at the 65 DAP (A), 95 DAP (B), and 120 DAP (C) stages**. Different letters on bars represent statistically significant differences (*P* < 0.05) for a given stage.

Figure S5 **Pentose accumulation in leaves of diverse maize and sorghum hybrids at the 65 DAP (A), 95 DAP (B), and 120 DAP (C) stages**. Different letters on bars represent statistically significant differences (*P* < 0.05) for a given stage.
