# Supplementary figures and images for: Stover Composition in Maize and Sorghum Reveals Remarkable Genetic Variation and Plasticity for Carbohydrate Accumulation
Source: Front Plant Sci. 2016 Jun 8;7:822. doi: 10.3389/fpls.2016.00822 (PMC4896940; doi:10.3389/fpls.2016.00822)

Figure S1

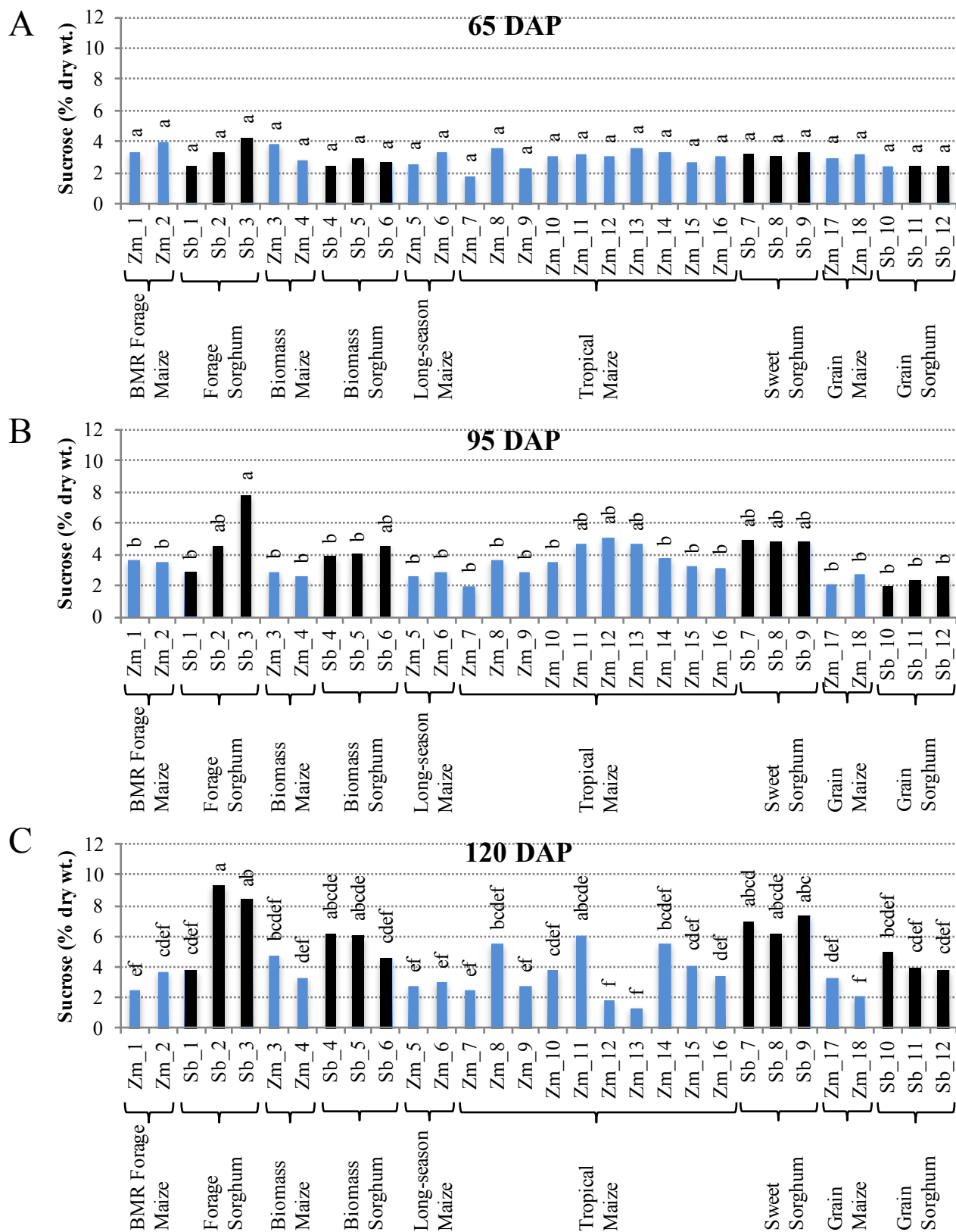

Supplement: Figure S1 — Sucrose accumulation in leaves of diverse maize and sorghum hybrids at the 65 DAP (A), 95 DAP (B), and 120 DAP (C) stages. Different letters on bars represent statistically significant differences (P < 0.05) for a given stage. [file Image1.pdf]

Figure S2

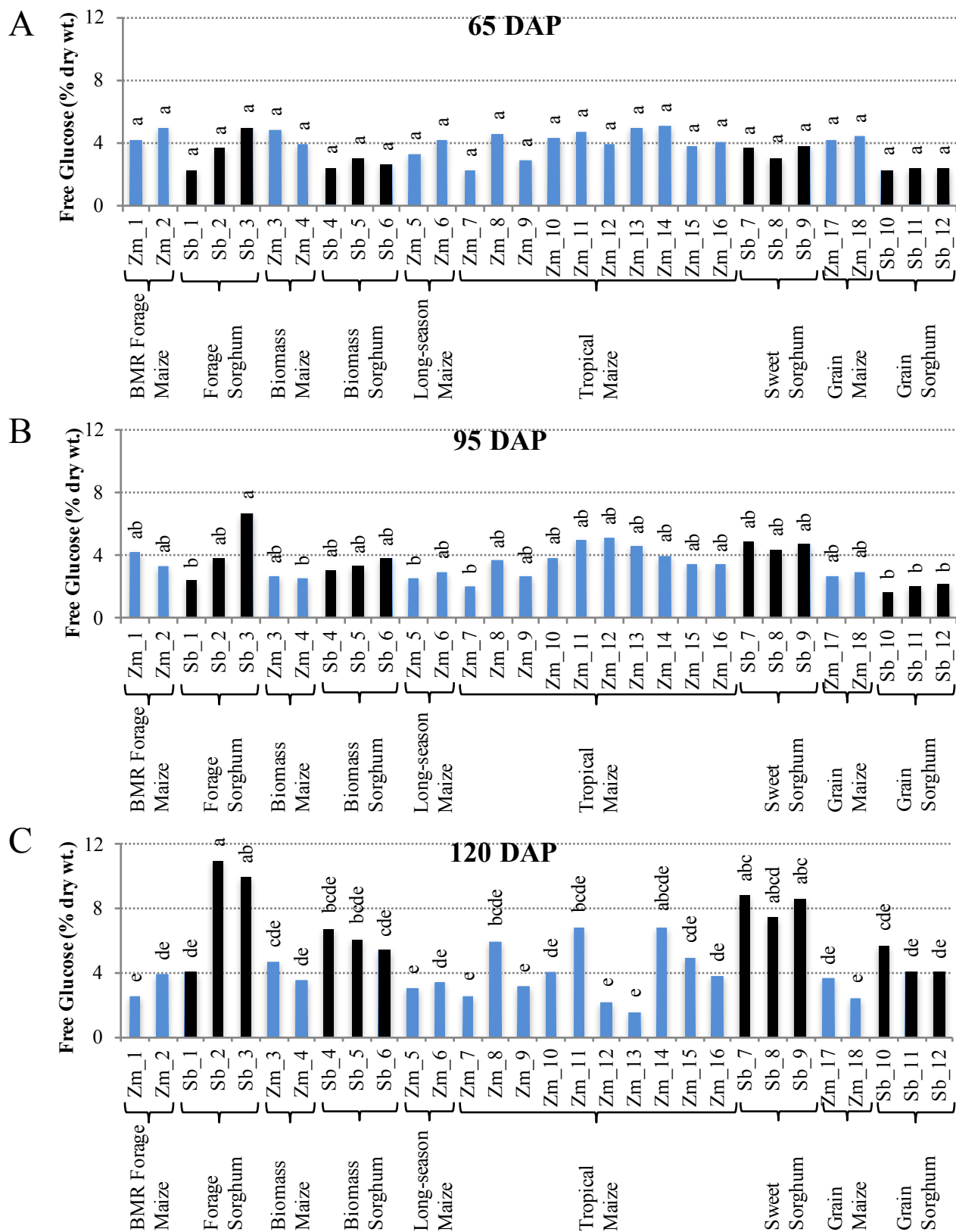

Supplement: Figure S2 — Free glucose accumulation in leaves of diverse maize and sorghum hybrids at the 65 DAP (A), 95 DAP (B), and 120 DAP (C) stages. Different letters on bars represent statistically significant differences (P < 0.05) for a given stage. [file Image2.pdf]

Figure S3

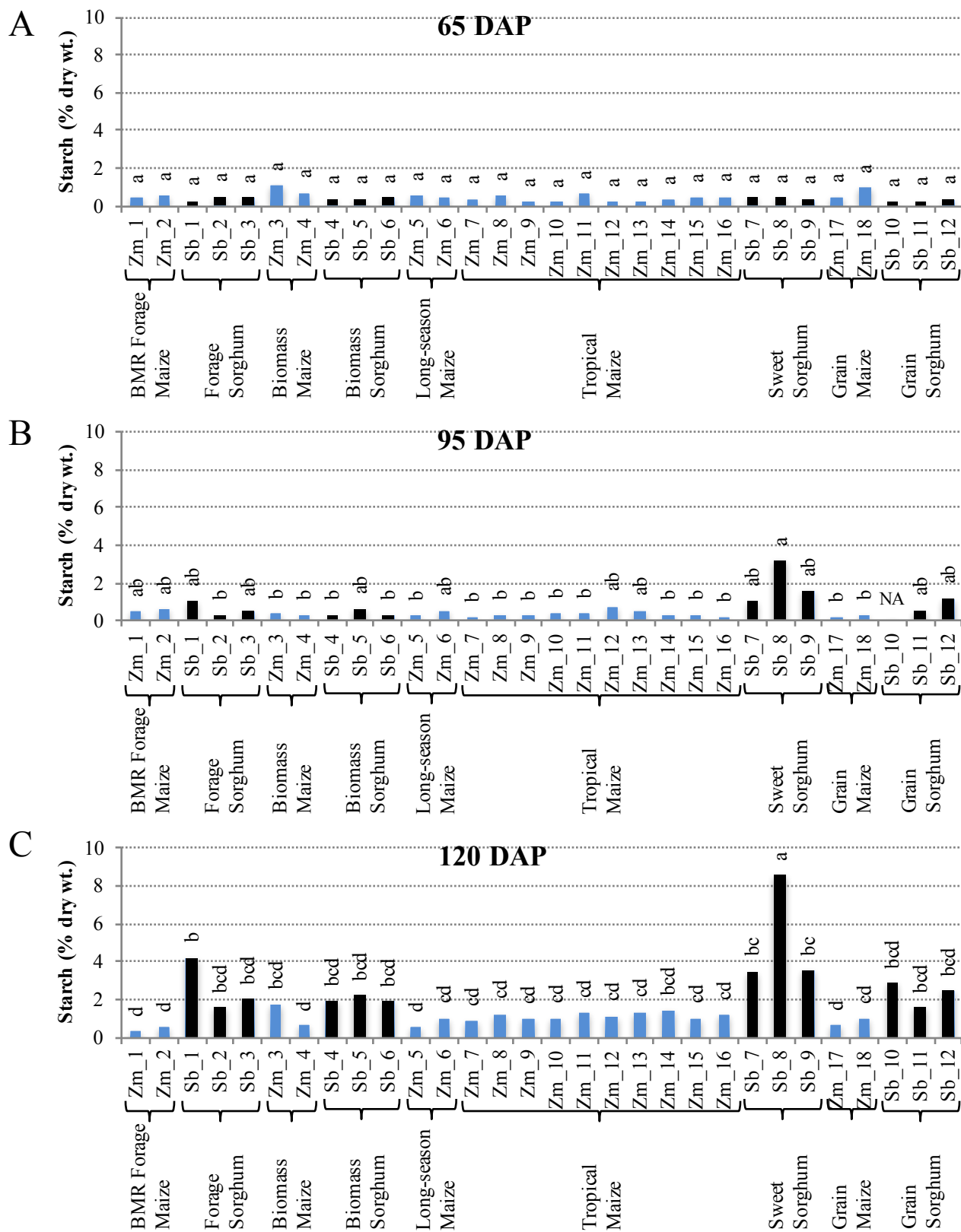

Supplement: Figure S3 — Starch accumulation in internodes of diverse maize and sorghum hybrids at the 65 DAP (A), 95 DAP (B), and 120 DAP (C) stages. NA, no data available. Different letters on bars represent statistically significant differences (P < 0.05) for a given stage. [file Image3.pdf]

Figure S4

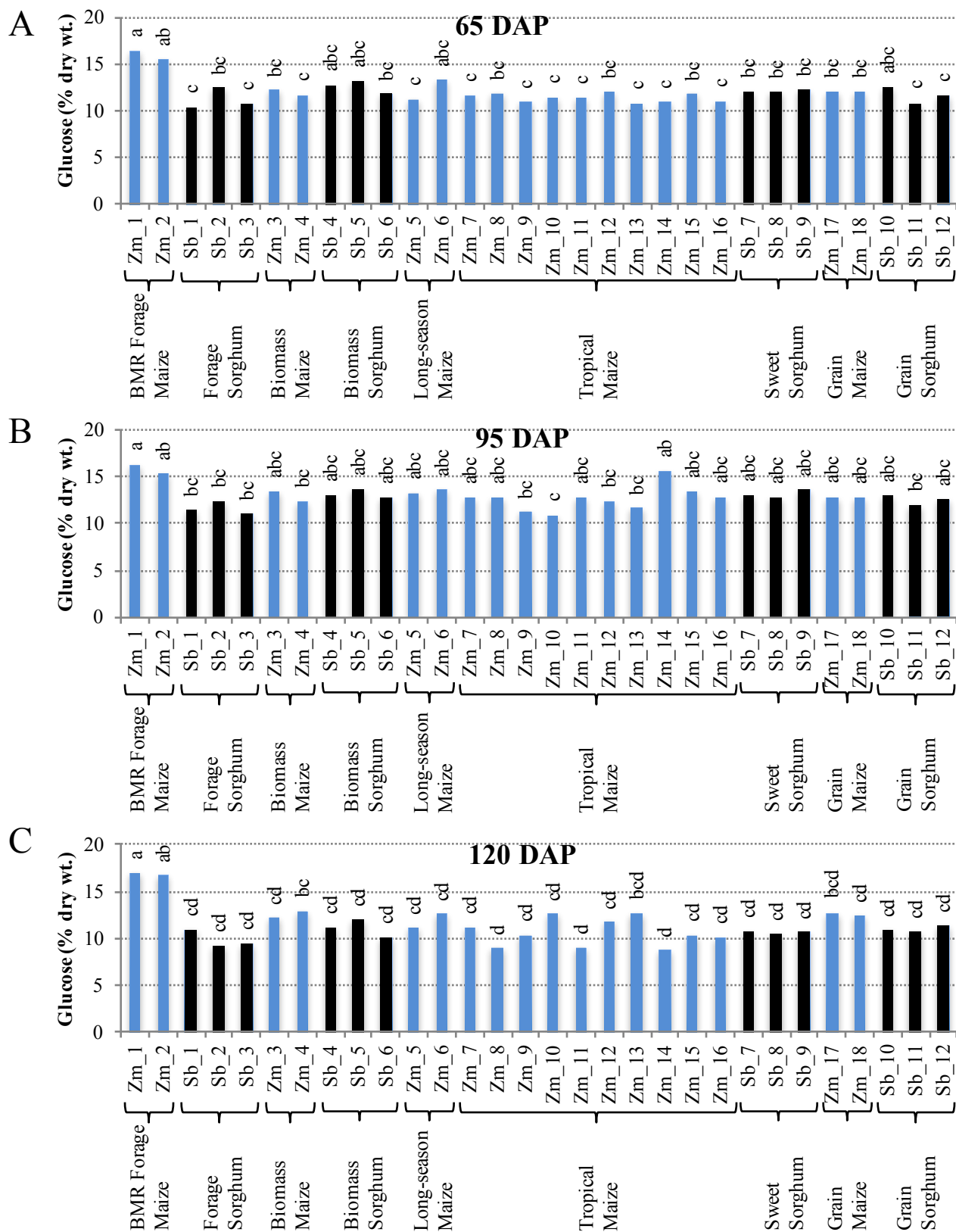

Supplement: Figure S4 — Glucose accumulation in leaves of diverse maize and sorghum hybrids at the 65 DAP (A), 95 DAP (B), and 120 DAP (C) stages. Different letters on bars represent statistically significant differences (P < 0.05) for a given stage. [file Image4.pdf]

Figure S5

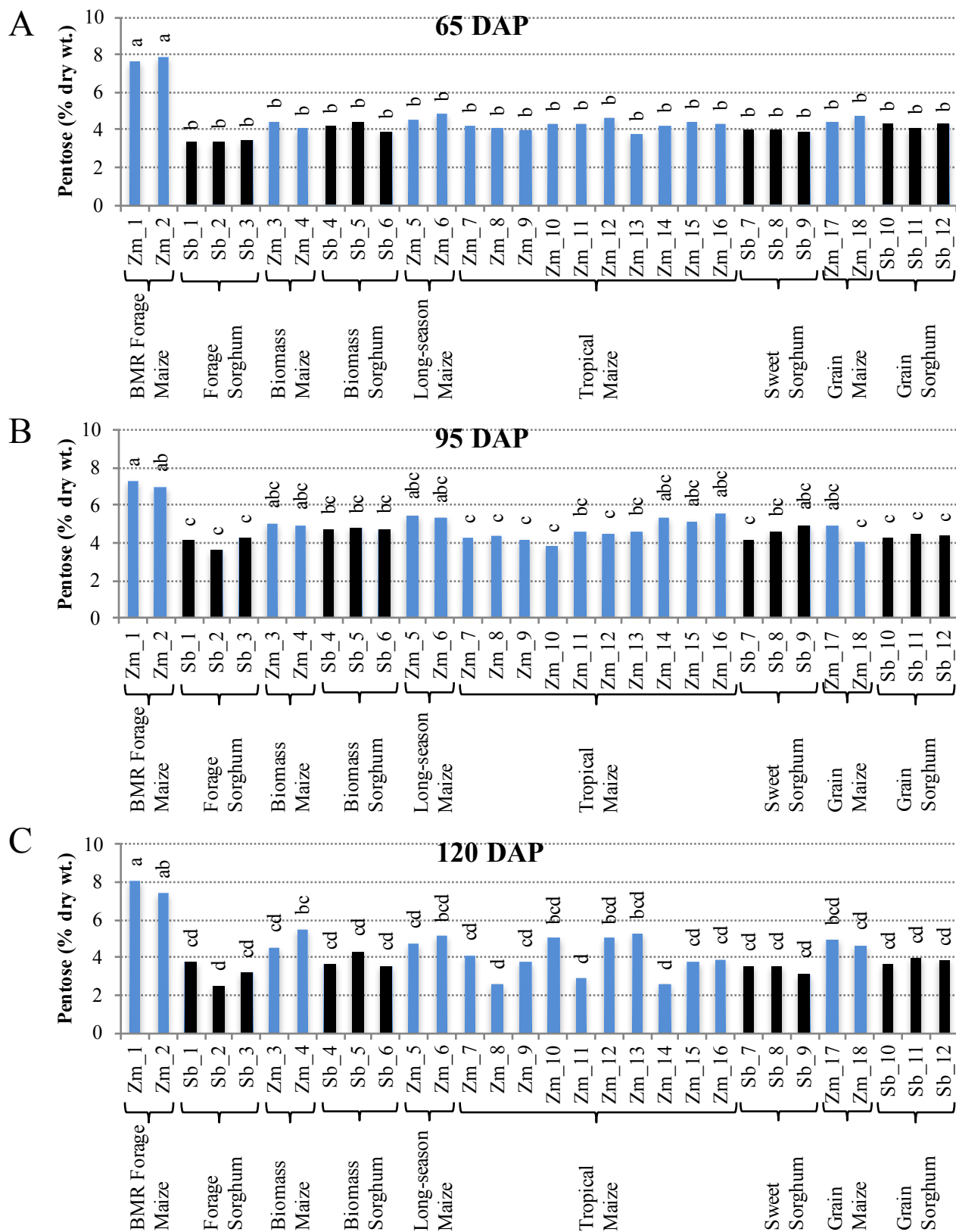

Supplement: Figure S5 — Pentose accumulation in leaves of diverse maize and sorghum hybrids at the 65 DAP (A), 95 DAP (B), and 120 DAP (C) stages. Different letters on bars represent statistically significant differences (P < 0.05) for a given stage. [file Image5.pdf]
