# Supplementary material for: Stover Composition in Maize and Sorghum Reveals Remarkable Genetic Variation and Plasticity for Carbohydrate Accumulation
Source: Front Plant Sci. 2016 Jun 8;7:822. doi: 10.3389/fpls.2016.00822 (PMC4896940; doi:10.3389/fpls.2016.00822)
Supplement: Supplementary file 6 [file DataSheet1.PDF]

Stover Composition in Maize and Sorghum Reveals Remarkable Plasticity for Carbohydrate Accumulation

Rajandeep S. Sekhon[1], Matthew W. Breitzman[2, 3], Renato Rodrigues[4], Nicholas Santoro[5], William L. Rooney[6], Natalia de Leon[2, 3], Shawn M. Kaeppler[2, 3, *]

1 Department of Genetics and Biochemistry, Clemson University, Clemson, SC, USA
2 Department of Agronomy, University of Wisconsin, Madison, WI, USA
3 DOE Great Lakes Bioenergy Research Center, University of Wisconsin, Madison, WI, USA
4 Institute of Mathematics and Statistics, Federal University of Goiás, Campus II, Goiânia, GO, Brazil
5 DOE Great Lakes Bioenergy Research Center, Michigan State University, East Lansing, MI, USA
6 Department of Soil and Crop Sciences, Texas A&M University, College Station, Texas, USA

Correspondence:

Shawn Kaeppler

smkaeppl@wisc.edu

SUPPLEMENTARY FIGURES

Figure S1: Sucrose accumulation in leaves of diverse maize and sorghum hybrids at the 65 DAP (A), 95 DAP (B), and 120DAP (C) stages. Different letters on bars represent statistically significant differences (P<0.05) for a given stage.

Figure S2: Free glucose accumulation in leaves of diverse maize and sorghum hybrids at the 65 DAP (A), 95 DAP (B), and 120DAP (C) stages. Different letters on bars represent statistically significant differences (P<0.05) for a given stage.

Figure S3: Starch accumulation in internodes of diverse maize and sorghum hybrids at the 65 DAP (A), 95 DAP (B), and 120DAP (C) stages. NA, no data available. Different letters on bars represent statistically significant differences (P<0.05) for a given stage.

Figure S4: Glucose accumulation in leaves of diverse maize and sorghum hybrids at the 65 DAP (A), 95 DAP (B), and 120DAP (C) stages. Different letters on bars represent statistically significant differences (P<0.05) for a given stage.

Figure S5: Pentose accumulation in leaves of diverse maize and sorghum hybrids at the 65 DAP (A), 95 DAP (B), and 120DAP (C) stages. Different letters on bars represent statistically significant differences (P<0.05) for a given stage.
